# The experiences of a structured pelvic floor rehabilitation program in colorectal cancer survivors with low anterior resection syndrome: A qualitative study

**DOI:** 10.1007/s00520-026-10892-8

**Published:** 2026-06-26

**Authors:** Kin Yin Carol Chan, Sarah E. Ratcliffe, Gemma Collett, Janindra Warusavitarne, Michael Suen, Susan Coulson, Janette L. Vardy

**Affiliations:** 1 Concord Cancer Centre, Concord Repatriation General Hospital, Sydney, Australia; 2 Faculty of Medicine and Health, University of Sydney, Sydney, Australia; 3 Department of Colorectal Surgery, Concord Repatriation General Hospital, Sydney, Australia; 4 St. Mark’s Hospital, London, North West England; 5 Australian Research Centre for Cancer Survivorship, University of New South Wales, Sydney, 2052 Australia

**Keywords:** Survivorship, Colorectal cancer, Low anterior resection syndrome, Pelvic floor rehabilitation, Symptom management, Behaviour change, Self-management, Feasibility, Qualitative

## Abstract

**Background:**

Low anterior resection syndrome (LARS) is a common survivorship challenge after colorectal cancer (CRC), affecting bowel function, psychosocial health, and daily living. Pelvic floor rehabilitation (PFR) is a supportive approach for bowel function recovery, but little is known about survivors’ experiences of or behaviour change processes when participating in a structured PFR program.

**Aim:**

This study explored CRC survivors’ experiences of LARS and structured PFR with focus on satisfaction, supportive care needs, and behaviour change processes.

**Design:**

This was a cross-sectional qualitative study. Analysis employed a hybrid inductive-deductive approach to theme development and data mapping with Symptom Management Theory (SMT) and the COM-B model.

**Setting/participants:**

Fourteen participants with LARS who had completed the PFR program completed a self-administered paper satisfaction survey and semi-structured telephone interview.

**Results:**

All participants rated their experiences as good to excellent on a 6-point Likert scale. Three themes related to participants’ LARS experience and PFR program participation were generated: (1) living with unpredictable LARS; (2) a desire for quality information, timely education, and individualised multimodal support; and (3) regaining function and control through structured rehabilitation. Processes of behaviour change were as follows: (1) expectation management; (2) gaining ability to manage; (3) self-efficacy and habit consolidation; and (4) re-evaluation and relapse management. Mapping to SMT and COM-B informed development of a new model, the empowered behavioural adaptation process (EBAP).

**Conclusions:**

LARS imposes considerable physical and psychosocial burdens on CRC survivors, worsened by unmet informational needs and fragmented support. Behaviour change and self-management theories explain how guided, supportive care and individualised multimodal PFR through an empowered behavioural adaptation process, support self-efficacy and long-term management of LARS in CRC survivors.

**Supplementary Information:**

The online version contains supplementary material available at 10.1007/s00520-026-10892-8.

## Introduction

Colorectal cancer (CRC) is one of the top five most commonly diagnosed cancers in Australia over the past decade [1]. Over half of CRC cases involve sigmoid and rectal cancers. Surgery remains the primary treatment, with 67% of patients undergoing anterior resection [2]. Depending on cancer stage, neoadjuvant and adjuvant therapies are offered to optimise oncological outcomes. Despite advances in management, approximately 40% of CRC survivors persistently experience symptoms of low anterior resection syndrome (LARS) from curative-intent surgery and chemoradiotherapy [3].

Low anterior resection syndrome (LARS) is characterised by one or more bowel symptoms, such as urgency, incontinence, and frequent or difficult bowel movements after surgery and chemoradiotherapy [4]. Impaired bowel function disrupts daily life and imposes an often underestimated psychological burden on survivors [5–7]. Recurring patterns observed in cancer survivors’ experiences include, unpredictability of bowel function affecting both continence and evacuation and unrealistic expectations of full bowel recovery causing ongoing psychological distress [8]. Existing patient-reported outcome measures (PROMS), such as LARS score [9] and Memorial Sloan Kettering Cancer Center–Bowel Function Instrument (MSKCC-BFI) [10], focus on bowel dysfunction symptoms but may underestimate this burden [11–14].

Functional recovery is a complex, prolonged process that requires a multimodal approach to manage the chronic nature of LARS [15, 16]. Yet, many CRC survivors are left to navigate these challenges with limited guidance, fragmented follow-up, and unrealistic expectations about returning to pre-treatment bowel function and social participation [17].

Pelvic floor rehabilitation (PFR), which includes pelvic floor muscle training, biofeedback (sensory, audio, visual), and education, has shown early promise as a treatment option for LARS [18–20]. However, trial variability with different protocols and outcome measures limits the development of effective, targeted LARS management programs [21]. Additionally, feasibility evidence for Australian healthcare system outpatient settings remains limited. Little is known about whether structured and guided PFR supports CRC survivors with LARS to manage bowel symptoms and improve quality of life (QOL) through behaviour change and self-efficacy.

Symptom Management Theory (SMT) and the Capability, Opportunity, Motivation–Behaviour (COM-B) model of behaviour change provide insights into understanding the multifaceted impacts of LARS on physical symptoms, psychological readiness, capability, and system-level barriers [22–24]. System-level barriers, including insufficient recovery planning information, low LARS awareness among clinicians, and lack of coordinated pathways for supportive care and rehabilitation, undermine patient preparedness and functional outcomes [25–27]. Need-based supportive strategies, including structured and supervised rehabilitation programs, support cancer survivors through behavioural adaptation essential for self-efficacy and sustainable improvement [28].

This study explored whether outpatient, physiotherapist-led PFR is a practical and supportive approach for CRC survivors living with LARS. By integrating behavioural science into clinical rehabilitation, this study seeks to inform more responsive, patient-centred care in cancer survivorship that goes beyond managing symptoms to promote patient self-efficacy in recovery decisions and support the restoration of life roles. Specific aims included the following: (1) assess satisfaction of structured PFR in a clinical setting; (2) explore survivors’ experiences and understanding of LARS; (3) identify supportive care needs of CRC survivors; and (4) determine the impact of PFR and its potential to facilitate behaviour change through expectation management, internal adaptation, and ongoing self-management.

## Methods

This was a cross-sectional qualitative study conducted as part of a feasibility project for a structured, physiotherapy-led PFR program for LARS after CRC surgery and treatment. Reporting was guided by the Consolidated Criteria for Reporting Qualitative Research (COREQ) checklist [29].

Participants were recruited from a PFR feasibility project conducted at a New South Wales outpatient clinic, Australia [30]. Participants were over 18 years old, had undergone sphincter-preserving anterior resection for CRC, experienced new onset of bowel symptoms defined as LARS score > 20 (score range 0–42; categorised as No LARS [0–20], minor LARS [21, 29], major LARS [30–42]) [31], and at least 6 months post-bowel continuity restoration.

The PFR program was designed for LARS and delivered by a pelvic floor dysfunction-trained physiotherapist over 10 weekly 60-min sessions. The first session incorporated education on pelvic floor and anorectal anatomy, LARS after CRC surgery and management strategies (including diet, optimal toileting and bladder/bowel habits, and correct pelvic floor muscle activation) to address storage and evacuation symptoms, mostly reported as urgency, frequency, and incontinence. Educational handouts were provided. A weekly supervised session consisted of pelvic floor muscle training with components of anorectal coordination, sensory awareness and defaecatory control using biofeedback. Participants also completed an individualised home exercise program with a bowel diary, which was reviewed weekly to monitor adherence and guide progression.

After completing the PFR program, participants were invited to evaluate the program via a self-administered satisfaction survey and semi-structured telephone exit interview (Supplementary file 1). Interviews were conducted within 1–2 weeks following completion of the PFR program. All participants provided written consent. Data collection continued until saturation or all participants completed interviews.

Interviews explored three key areas: (1) impact of LARS on daily lives; (2) perceptions of PFR in alleviating symptoms and improving QOL; and (3) overall satisfaction with PFR (see Supplementary file 1). Lead author (KYCC) developed interview questions and flexible prompts guided by Symptom Management Theory (SMT)[23]. An independent researcher (GC), experienced in qualitative interviewing and with no prior involvement in the intervention, conducted the interviews in English. One participant requested translation support from a family member with responses paraphrased rather than a professional interpreter. While efforts were made to ensure accurate communication and to preserve the participant’s meaning, the use of a non-professional interpreter may have influenced the phrasing and level of detail in responses. Interviews were audio-recorded and transcribed verbatim using TRINT audio transcription software [32].

Quantitative data were analysed using SPSS version 26 [33]. Qualitative data were analysed using thematic analysis with a hybrid inductive-deductive approach [34] using NVivo 14 [35], Microsoft PowerPoint [36], and digital whiteboards. Iterative development and refining of concepts, patterns, and themes continued until consensus on thematic interpretations was reached.

To situate the findings within established theoretical frameworks relevant to symptom management and behaviour change, codes were mapped onto the elements of Symptom Management Theory (SMT) and the Capability, Opportunity, Motivation–Behaviour (COM-B) model [23, 24] (see Supplementary File 2). While SMT and COM-B models guided interpretation, theme development remained inductive and grounded in participants’ accounts. Further explanation of methodological rigour is provided in Supplementary File 3.

## Results

Fourteen CRC survivors completed the 10-week PFR program, including an exit survey and an interview. Mean age was 62.1 years (range 44–81), 85.7% had rectal cancer, and 71.4% had an ultralow anterior resection and a temporary stoma. All participants rated the program as good to excellent (see Table 1 for demographic and satisfaction data).

**Table 1:** Demographic information and PFR satisfaction of participants

Participants’ demographics (*n* = 14)	Mean (range)/% (*N*)
Age (years)	62.1 (44–81)
Sex	
Female	50.0% (7)
Male	50.0% (7)
Marital status	
Married	64.3% (9)
Widowed	21.4% (3)
Divorced	14.2% (2)
Employment	
Employed full time	14.3% (2)
Self-employed	28.6% (4)
Unemployed	7.1% (1)
Retired	50.0% (7)
Cancer stage	
Stage I	21.4% (3)
Stage II	35.7% (5)
Stage III	42.9% (6)
Cancer location	
Sigmoid	14.3% (2)
Rectum	85.7% (12)
Procedure	
High anterior resection	7.1% (1)
Low anterior resection	21.4% (3)
Ultralow anterior resection	71.4% (10)
Temporary stoma (yes)	71.4% (10)
Stoma duration (months)	4.6 (2–10)
Time since bowel continuity restoration (months)	21.0 (7–60)
Neoadjuvant therapy	
Long course chemoradiotherapy	21.4% (3)
Short course radiotherapy	7.1% (1)
No neoadjuvant therapy	71.4% (10)
Adjuvant therapy (yes)	42.9% (6)
LARS score^a^	32.9 (24–41)
LARS category^a^	
Minor	42.9% (6)
Major	57.1% (8)
PFR satisfaction–overall^b^	
Excellent	64.3% (9)
Very good	28.6% (4)
Good	7.1% (1)
PFR satisfaction–psychological impact^c^	
Received support was adequate	
Strongly agree	78.6% (11)
Agree	21.4% (3)
Confidence regained	
Strongly agree	64.3% (9)
Agree	35.7% (5)
PFR satisfaction–knowledge advancement^c^	
Physiotherapist who delivered PFR was knowledgeable in answering questions	
Strongly agree	85.7% (12)
Agree	14.3% (2)
Opportunity given for discussion	
Strongly agree	71.4% (10)
Agree	28.6% (4)
PFR satisfaction–practicality^c^	
Information comprehensibility	
Strongly agree	64.3% (9)
Agree	35.7% (5)
Information was useful	
Strongly agree	85.7% (12)
Agree	14.3% (2)
Exercises were clear and useful	
Strongly agree	57.1% (8)
Agree	42.9% (6)
Dietary advice	
Strongly agree	50.0% (7)
Agree	50.0% (7)
Clinic accessibility	
Strongly agree	57.1% (8)
Agree	42.9% (6)

^a^LARS symptom severity: range, 0–42; 0–20 = no 21–29 = minor, 30–42 = major

^b^PFR satisfaction–overall: rating on a 6-point Likert scale (very poor, poor, average, good, very good, excellent)

^c^PFR satisfaction–psychological impact, knowledge advancement, practicality: responding on a 4-point Likert scale (strongly agree, agree, disagree, strongly disagree)

**Fig. 1:**
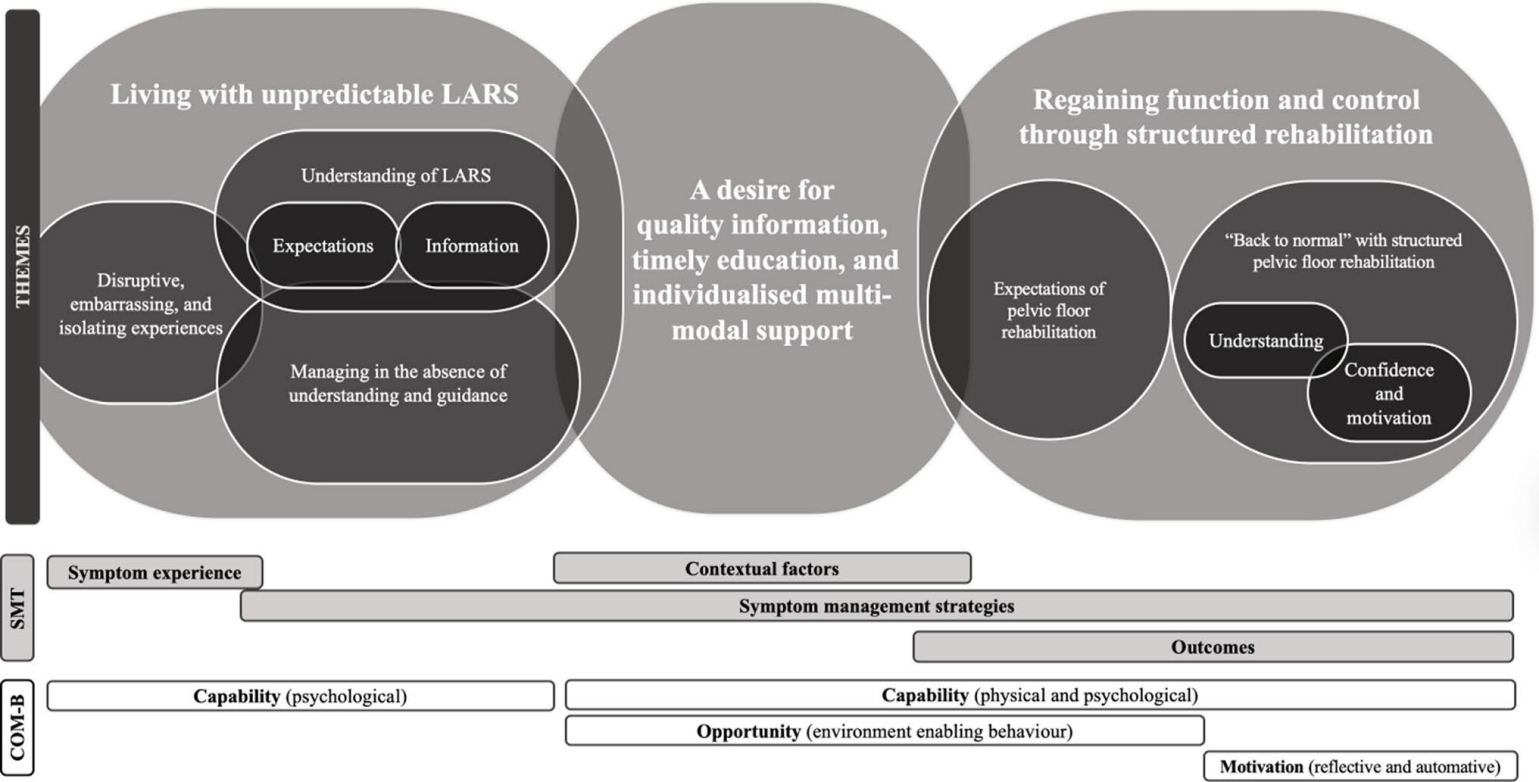
Visualisation of themes developed during analysis, layered with SMT and COM-B. Dark toned ovals represent themes and subthemes developed from this dataset; grey boxes represent components of SMT; white boxes represent components of COM-B

Thematic analysis developed three themes related to participants’ experiences with LARS and PFR participation: (1) living with unpredictable LARS; (2) a desire for quality information, timely education, and individualised multimodal support; and (3) regaining function and control through structured rehabilitation (Fig. 1). Supplementary File 4 presents additional quotes.

### Theme 1: Living with unpredictable LARS

Participants described living with LARS as difficult and disruptive. They demonstrated varying levels of understanding of LARS, often describing the physical and psychological impacts as more severe than anticipated, prompting adjustments to expectations and modifications in management of persistent LARS symptoms. Knowledge of LARS was linked with experiences and limited information provided by healthcare professionals. This was reported to influence expectations for pelvic floor rehabilitation in aiding bowel function recovery.

### Disruptive, embarrassing, and isolating experiences

All participants reported experiencing LARS-related symptoms, including increased bowel frequency, urgency, leakage, pain, and change in stool consistency. Bowel routines were frequently described as “erratic and frequent” [P1], with some participants needing to use the toilet “seven to eight times a day” [P8] or “missing the toilet many times… [and] soiling my underwear” [P2]. LARS symptoms also occurred overnight, compounding participants’ distress.

The disruptive nature of LARS symptoms was closely tied to emotional and social consequences, including social withdrawal and reluctance to engage in everyday activities. Participants expressed frustration, fear, embarrassment, diminished confidence, and a lack of control. Feeling stigmatised or dismissed by others, including family members, exacerbated their sense of isolation.

embarrassingly enough, I had a couple of accidents… I got to the stage where I was worried about going out, and I would make sure that I was going to a place where there were toilets. [P5]

### Understanding of LARS

Participants’ experiences and knowledge of LARS influenced their initial understanding of recovery. Quality of information received was linked with the perceived impact of LARS. Absent or incomplete information impacted medical decision-making, with one participant sharing, “I think if you told people the extreme of it, people would be very cautious about having surgery.” [P14].

#### Expectations and realisations of living with LARS

While participants initially believed that bowel function would return to their pre-surgery state without intervention, some accepted that full recovery was unlikely, acknowledging they were “never going to be 100%” [P3]. The expectation of recovered bowel function was often not grounded in a realistic “understanding of how the bowels work” [P11], particularly regarding physiological impacts of surgery and treatment. Participants were surprised at the extent of muscle weakness and persistence of symptoms.

I didn’t really appreciate how weak the muscles have become [since surgery]… I just thought, ‘oh yes, I’m a perfectly healthy person. I’ve never had any trouble before. So, you know, why should I be weak [now]? [P1]

While some participants attributed worsening symptoms to ageing, the lingering impact of cancer treatment was widely considered the primary cause. Several participants indicated the burden of LARS was preferable to a permanent stoma, which was perceived as more stigmatising and psychologically distressing.

They wanted me to have a stoma bag for life, and I said no… I would rather die than have that. [P9]

#### Delayed, inconsistent, and absent LARS information

Participants consistently reported limited attention to potential long-term bowel dysfunction and its impact on QOL. Clinical consultations focused on cancer treatment plans and surgical preparation and lacked comprehensive LARS education. Some described information as superficial, overly complex, or generalised, noting clinicians had limited time. Participants felt postoperative bowel issues were a secondary consideration for clinicians, leading to feelings of neglect regarding their ongoing recovery and care needs.

Before the surgery I didn’t know any of this was going to happen. And I don’t think he’s worried about that. It’s about fixing the problem. [P4]

Some encountered prolonged gaps in communication with information on LARS first received months postoperatively. Quality and timing of information was described as “rude” [P6] and evoked feelings of frustration, confusion, indignation, and helplessness, largely attributed to the ambiguity, inconsistency, and contradictions in the information offered. A particular concern and source of distress was the lack of clear expectations regarding the recovery timeline and likelihood of functional restoration.

The information is different depending on who you talk to. It’s different from the nurse or from the doctor. [P9]

### Managing in the absence of understanding and guidance

Participants described their experiences with LARS as differing from what they were told to expect. They managed by adjusting their expectations for living with LARS and, in the absence of professional guidance, adopting various passive and short-term solutions to regain control of their lives. Many described feeling helpless and uncertain about how to manage their symptoms; often resulting in inaction.

I didn’t do anything because I didn’t know what to do. [P11]

Initially participants were encouraged by healthcare professionals’ optimistic predictions but when symptoms did not improve, they adjusted their expectations and adopted pragmatic, symptom-focused management strategies.

It took quite a few months to get used to [the change]… I realised that it’ll never be the same again. [P5]

Most participants relied on self-devised coping strategies for symptom management, often involving trial and error. While sometimes helpful, these approaches often produced inconsistent outcomes. To minimise disruption from unpredictable bowels, personalised routines were developed.

Before I used to go out, if I have an appointment or anything to do in the morning. Yeah, I didn’t used to have any breakfast at all. [P6]

#### Theme 2: A desire for quality information, timely education, and individualised multi-modal support

In response to a lack of timely, comprehensive, and consistent information about bowel function and cancer treatment, participants expressed desire for more in-depth discussions about bowel function and recovery expectations, alongside adequate time and support to process information.

so preparing somebody for the [stoma] reversal would be a great help. And possibly getting them started on the program like this once the reversal is done, or even before so that people can then become aware. [P5]

Participants wanted accessible, evidence-based solutions to address symptoms and restore a sense of normalcy and functional control.

I was experiencing, I would say debilitating [LARS] symptoms, and I wanted to try anything possible that could kind of help with that. [P14]

Participants emphasised the need for extended, multidisciplinary care beyond the acute treatment phase, noting gaps in continuity of care after hospital discharge, advocating for a structured and supportive rehabilitation framework.

I think sometimes a program [like PFR] in extended form would help you understand [LARS]. [P2] 

#### Theme 3: Regaining function and control through structured rehabilitation

Participants highlighted the contribution of a physiotherapist and the PFR program as central to their recovery experience. Entering the program, they had limited understanding and cautious optimism for PFR. Participation was noted to shift LARS management strategies towards more informed and intentional approaches and prompt sustainable improvements in function and QOL.

### Expectations of PFR

Participants often entered the PFR program with minimal knowledge and vague expectations about its purpose. Many reported PFR had not been discussed previously. Several participants viewed PFR as a final opportunity for improvement, adopting a mindset of, “nothing to lose.” [P1]. Some managed their expectations to avoid disappointment, framing the intervention as a tool for coping rather than a cure.

I don’t know what I really expected other than just to learn how to cope better… my surgeon believed it would be a good study for me with the symptoms I am experiencing post-cancer. [P13]

### “Back to normal” with structured PFR

Participants reported “dramatic improvement” [P3] in their bowel symptoms, functionality, understanding of LARS, sense of control, and overall well-being as the program progressed. It did not resolve all symptoms, but participants saw it as an important step toward lessening LARS disruption in daily life and supporting their function to, “come back to normal” [P7], or be, “85–90% better than it was” [P5]. By the end of the PFR program, many participants had resumed activities they had previously avoided, including social outings and travel.

Understanding their body was regarded as key to a more sustainable, confident, and motivated way forward following PFR. Participants emphasised the value of the supervised, multimodal nature of the PFR program, incorporating functional training, biofeedback, and education to gain an understanding of their body, and provide confidence and motivation to move forward.

[the physiotherapist] explained a lot of things to me… and made me more realise what the problem I have and what I should do. [P12]

#### Understanding my body

All participants highlighted pelvic floor muscle training as key to their functional bowel improvement. With tailored instructions, they gained an understanding of the connection between pelvic floor function and bowel control. The exercises improved body awareness and enabled the strengthening of muscles previously not recognised as relevant.

Physio gave me a visual of kind of like the cradle kind of thing and like the pelvic floor and the different layers of everything. So, I understand a lot more about how it works, [P13]

Real-time biofeedback was instrumental in helping participants understand and correct their technique. Visual (ultrasound) and sensory (rectal balloon) tools, along with therapist feedback, supported cognitive-motor learning and exposed maladaptive habits participants had unknowingly developed.

I actually saw the ultrasound and I saw what was happening when I contracted. When I did the pelvic floor exercise, I saw things happening inside of me that spoke major volumes to me. [P5]

Alongside biofeedback, information about pelvic floor muscles and bowel function anatomy helped simplify complex concepts into understandable details, leading to a better understanding of bowel function. The combination of pelvic floor exercises and bowel retraining supported improved understanding of how diet impacts bowel symptoms. This led to more confident and consistent self-management, such as food choices and meal timing.

We changed some things in my diet, and it made a big difference. I don’t really have dairy anymore. [P13]

#### Moving forward sustainably, with confidence and motivation

Participants noted a shift from relying on passive strategies to confidently identifying symptom triggers and using targeted techniques to improve bowel and muscle function. Many reported reduced dependence on incontinence products and medications, along with increased trust in their ability to self-manage.

I feel like I was listening to my body’s cues and almost retraining the muscles, to allow me to hold on for longer. [P14]

Participants shared how addressing functional symptoms and counselling addressed concerns, reducing anxiety and daily uncertainty. The ability to anticipate and manage symptoms reduced stress and anxiety, restored confidence, and provided a sense of control. Social activities and travel became more feasible.

I am today without any pull-ups on, I used to use those pants under underpants, but because I have control after the program, because I didn’t have to use them… I feel confident going out now. [P2]

As participants progressed through the PFR program, many reported “You are motivated to keep going” [P2] due to symptom relief, self-efficacy, and personal growth. A trusting relationship with the therapist was key in fostering motivation and adherence, encouraging ownership of their health. This motivation translated into a proactive mindset shift, with some planning long-term wellness strategies, such as continued exercise and integration of pelvic floor routines into daily life.

So the next thing I am going to do is join the gym. I’ll be going to start exercising more, and more pelvic exercises at the gym. [P9]

#### Mapping thematic results to Symptom Management Theory and COM-B

Table 2 provides a summary of how the inductively developed themes map to Symptom Management Theory (SMT) [23] and the Capability, Opportunity, Motivation–Behaviour (COM-B) model [24].

**Table 2:** Matrix of themes and Symptom Management Theory (SMT) and COM-B models

**SMT domain or COM-B component**		**1. Living with unpredictable LARS**	1.1 Disruptive, embarrassing, and isolating experiences	1.2 Understanding of LARS	*1.2.1 Expectations and realisation of living with LARS*	*1.2.2 Delayed inconsistent and absent LARS information*	1.3 Managing in the absence of understanding and guidance	**2. A desire for quality information, timely education, and individualised multimodal support**	**3. Regaining function and control through structured rehabilitation**	3.1 Expectations of PFR	3.2 “Back to normal” with structured PFR	*3.2.1 Understanding my body*	*3.2.2 Moving forward sustainably, with confidence and motivation*
**Symptom experience**	Perception	x	x										
	Evaluation	x	x	x	x		x						
	Response	x					x	x					
**Components of symptom management strategies**		x					x	x	x				x
**Outcomes**		x					x		x		x		
**Environment**		x		x		x		x	x	x			
**Person**		x						x	x	x		x	
**Health and illness**									x		x		
**Adherence**									x		x		x
**Capability**	Physical								x		x		x
	Psychological	x	x	x	x		x	x	x		x	x	x
**Motivation**	Reflective								x		x		x
	Automatic								x		x		x
**Opportunity**	Physical	x		x		x			x	x			
	Social	x		x		x			x	x			
**Behaviour**		x					x		x		x		

Theme 1, *Living with unpredictable LARS*, reflects the symptom experience domain of SMT and psychological capability, motivation, and behaviour components of COM-B. Participants perceived and evaluated the impact of LARS on their physical, psychological, and social functions. LARS’ disruption to daily routines prompted (COM-B reflective motivation) early adaptive self-management (SMT symptom experience response; COM-B behaviour). Limited understanding of LARS hindered participants’ ability to recognise realistic recovery expectations (SMT symptom experience evaluation; COM-B psychological capability) and engage in rehabilitation (SMT symptom experience response).

Theme 2, *A desire for quality information, timely education, individualised multimodal support*, maps to the environment domain of SMT and opportunity component of COM-B, emphasising system-level barriers to symptom management. Participants reported inadequate preparation for recovery and insufficient, inconsistent information on LARS during their formal care pathways. Lack of individualised and LARS-specific education and delayed coordinated care, limited opportunity to effectively navigate development of LARS management behaviours.

Theme 3, *Regaining Function and Control Through Structured Rehabilitation*, aligns with the symptom management strategies and outcome domains of SMT. As participants progressed through PFR, they developed a proactive plan to re-establish routines and manage unpredictable bowel movements (SMT outcome). This behavioural shift (COM-B behaviour) was supported by skill acquisition and physiological control (COM-B physical capability), education and informed, guided learning (COM-B psychological capability), and goal-setting and perceived benefits (COM-B reflective motivation). Social reinforcement from established rapport and interaction with a physiotherapist facilitates social opportunities and motivation, leading to long-term behavioural change. Throughout PFR (SMT environment; COM-B opportunity) participants described increasing motivation (SMT personal context; COM-B automatic and reflective motivation) to adopt proactive approaches to managing LARS (SMT symptom management strategies), influenced by automatic and reflective processes during program participation.

## Discussion

This study examined the nuances of LARS’ impact on people’s daily lives and the role of PFR in managing LARS. Our findings further clarify CRC survivors’ limited understanding and unrealistic expectations of LARS, and provide new insights into their psychosocial experiences and symptom management in the absence of professional guidance. Participants’ reflections on PFR demonstrated that a structured, continuous, and multidisciplinary care model encourages behavioural changes for long-term management. It supports physical and psychosocial functioning, and is well received by CRC survivors with LARS. This supports the inclusion of PFR in the clinical pathway in survivorship care.

### Interpretation of key themes

Our findings align with Keane et al.’s (2020) LARS definition [4], which highlights its complexity in relation to an individual’s physical and psychosocial well-being. *Living with unpredictable LARS* highlights how this long-term complication of CRC treatment substantially affects people’s daily lives and challenges their psychosocial safety net [6]. As identified in this study and supported by previous research, these effects are often underestimated due to a focus on earlier phase care [5]. Our results demonstrate individuals living with LARS initially face considerable uncertainty and emotional strain trying to cope without professional support. The reactive strategies adopted reflect the underlying anxiety and uncertainty experienced by participants, particularly the fear of social embarrassment and loss of control in unfamiliar environments, often with inconsistent outcomes [36, 37].

Similar to Thomas (2019), we found a lack of clinician awareness and insufficient counselling about the long-term implications of LARS was central to the psychological burden of LARS [25]. This knowledge gap leaves many survivors unprepared for their new bowel routine, with physical and psychosocial responses shaped by their subjective experiences of living with these consequences [24, 37–39]. This suggests the need for setting realistic expectations to facilitate the psychological internalisation necessary for behavioural change [8, 40].

Although the current survivorship care model promotes an integrated, need-specific, multidisciplinary approach [41, 42], participants did not experience timely and personalised support during bowel function recovery. Communication gaps and dependence on a single clinician were common, limiting access to diverse expertise. This restricted participants’ ability to make informed decisions about their recovery options. Findings align with existing literature indicating a lack of LARS treatment guidelines, fragmented MDT supportive care, and unmet informational needs lead to suboptimal clinical practice [27, 43–45]. This undermines survivors’ preparedness for behavioural change, leaving them to navigate complex and chronic symptoms without evidence-based guidance.

A guided and needs-based care approach is essential for supporting behavioural adaptation in CRC survivors [46]. The results about *Regaining Function and Control Through Structured Rehabilitation* indicate a physiotherapist-led PFR program facilitates physical function and psychosocial recovery by providing education and psychological support. As per SMT [37] and COM-B [24], building physical and psychological capability alongside motivation, and providing physical, social, and cultural environments with opportunity, facilitates sustainable behavioural change for the adjustment and management of chronic conditions [24, 47].

### Empowered behavioural adaptation process model

Our thematic results highlight the challenges faced by CRC survivors and identify missed opportunities within the current cancer care model. The results highlight how struggles with physical symptoms and emotional burden are inseparable, and navigating everyday life requires sustainable change through psychosocial adjustment and behavioural adaptation. We propose that integrating behavioural science principles, such as expectation management, self-efficacy, and behavioural change, into survivorship care can better support cancer survivors in moving from uncertainty to confidence and from coping to thriving.

**Fig. 2:**
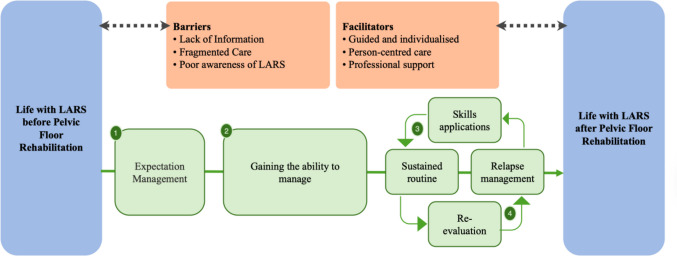
Empowered behavioural adaptation process (EBAP) in the context of PFR for people living with LARS.green boxes = in stage of process of adapting to live with LARS; blue = stage of management; orange = facilitators and barriers of management

The empowered behavioural adaptation process (EBAP; Fig. 2) addresses the temporal and relational complexities of survivorship in the context of LARS. EBAP emphasises the process of the following: (1) expectation management, involving psychological preparation for active rehabilitation and rebuilding confidence by setting realistic goals and normalising variability; (2) gaining ability to manage through therapist-guided skill development and meaning-making, offering opportunities and feedback to reinterpret, reframe, and regulate symptoms into manageable experiences; (3) self-efficacy and habit consolidation, where survivors apply skills into sustainable routines through situational cues and self-monitoring; and (4) re-evaluation and relapse management for maintaining long-term adaptation and resilience.

EBAP is grounded in behavioural science principles, integrating key constructs from SMT, COM-B, stress and coping, and self-efficacy. Inadequate information about LARS was a major barrier to functional recovery, and psychological capability and knowledge were closely linked to survivors’ ability to manage their recovery journey; hence, the principle of self-efficacy and its role in self-managed recovery [47, 48]. CRC survivors face biopsychosocial challenges from LARS that strain their pre-existing internal coping resources, such as resilience, and the external factors, like limited support or inconsistent professional guidance, that undermine their confidence in resuming normal life [49–51]. The existing model of stress and coping states that adaptive coping requires internal resources and external support [52], while social cognitive theory highlights self-efficacy as central to adaptation and behaviour change [53].

While the COM-B model [24] identifies capability, opportunity, and motivation as determinants for behaviour change, it does not fully capture the temporal and relational processes of recovery in survivorship with long-term functional disability. EBAP builds on COM-B by embedding these determinants within a sequential process, highlighting the role of clinician scaffolding and demonstrating that rebuilding capacity is an ongoing process. Importantly, EBAP provides a prescriptive mechanism to foster post-traumatic growth by sequentially connecting expectation management, proactive coping, and sustained self-efficacy to positive psychological adjustment after adversity [49]. Our findings indicate that a structured, therapist-supported, patient-centred program is more likely to facilitate a shift from a passive to a proactive mindset, enabling survivors to regain control and confidence in managing their bowel function [27, 54, 55].

EBAP acknowledges the complexity of living with LARS and outlines a process for sustainable change for QOL with LARS. This model is based on empirical data and established behavioural theories. With further testing and validation across a wider cancer population, EBAP offers a model for designing a PFR program that integrates physical recovery, and supports durable psychosocial and behavioural adaptation in CRC survivorship [56, 57].

### Strengths and limitations

This study offers insights into how LARS affects the lives of CRC survivors. It gives voice to survivors’ perspectives of LARS and a structured physiotherapist-led PFR program and uses established theoretical frameworks to interpret how behavioural change occurs through needs-specific interventions. Several limitations are acknowledged. The experiences captured might reflect a subgroup of more motivated individuals, with clinicians more engaged in the patient’s post-treatment recovery and more inclined to refer to PFR. Selection bias might be present in this sample, given their higher health literacy and greater self-advocacy in seeking medical information. The result interpretation could be influenced by positive-response bias, as participants’ beliefs about the consequences of action and emotional responses after completing PFR program, as well as social desirability or gratitude towards clinicians, may have led to more favourable reporting of experiences. These responses may differ from those of individuals who have never received similar treatment or from those of people with prior PFR under different protocols and structures. This bias should be considered when interpreting the findings, as the perspectives captured may differ from those of less-engaged patients.

Recruitment from a single metropolitan Australian centre may limit generalisability due to the potential under-representation of the broader population of CRC survivors experiencing LARS. Participants’ experiences might be influenced at various levels, including their demographics (e.g., health literacy, socioeconomic status, ability to access services), self-advocacy for treatment, and clinicians’ level of engagement in PFR, which can extend to health system resources. Although the findings should be interpreted within the context of a single-centre study, they offer a theoretical generalisability, meaning that the insights from SMT and COM-B behavioural framework, along with the proposed theory EBAP, can still inform clinical practice in other hospitals with similar resource constraints, clinical practices, and patient demographics. While a single-centre focus limits generalisability, the consistency of our findings with the broader literature [39, 40, 58] suggests that educational gaps are a widespread systemic issue. Consequently, these findings may be transferable to other hospital units with similar clinical practices and counselling protocols.

Using a family member as an interpreter may have affected data quality for that interview, including potential loss of nuance or meaning due to paraphrasing responses. Confidentiality concerns may have reduced willingness to disclose sensitive information. The absence of a professional interpreter may have affected the accuracy and consistency of translation. The research team’s expertise in CRC added valuable clinical context, enriching the interpretation and credibility of results and providing latent meaning and contextual grounding to ensure the theoretical sensitivity and clinical relevance of the themes. We acknowledge the potential for professional bias and assumptions of overlooking unfit data. Collaborative coding and ongoing reflexive discussion during analysis and write-up helped reduce risk of bias; nonetheless, future research could incorporate more interdisciplinary stakeholders, such as behavioural scientists and patient representatives, to enhance interpretation and challenge assumptions.

### Clinical implications and future direction

Findings highlight the need for timely, specific, and consistent LARS education and counselling to support psychological readiness for functional recovery after CRC treatment, recognising the variability and chronicity of LARS. Embedding structured, physiotherapist-led PFR into interdisciplinary and coordinated survivorship care is necessary for sustainable health outcomes. A national guideline for LARS rehabilitation is needed to raise clinicians’ awareness and ensure equitable access to healthcare for people with LARS. Future implementation research in broader settings, including diverse socioeconomic populations across multiple centres, would further inform the design and implementation of a survivor-centred model of LARS management.

## Conclusion

Our results indicate cancer survivors face physical and psychological challenges from LARS, including a loss of control and withdrawal from daily activities. Unmet informational needs, unrealistic recovery expectations, and fragmented support after CRC treatment contribute to the difficulty of living with LARS. Behaviour change and self-management theories help explain how guided, supportive care and individualised multimodal PFR support can enhance self-efficacy and support long-term management of LARS using an empowered behavioural adaptation process. Practice, policy, and future research should investigate the implementation of interdisciplinary, multimodal LARS management approaches into survivorship care in broader settings within clinical guidelines.

## Supplementary Information

Below is the link to the electronic supplementary material.

ESM 1 (DOCX 26.8 KB)

ESM 2 (DOCX 15.1 MB)

ESM 3 (DOCX 14.5 KB)

ESM 4 (DOCX 26.0 KB)

ESM 5 (DOCX 14.2 KB)

## Data Availability

The datasets generated during and/or analysed during the current study are available from the corresponding author on reasonable request. Portions of methodology and initial results were first presented at the Australian Physiotherapy Association Scientific Conference 2025 in Adelaide, Australia and have been expanded upon in this manuscript.
