# Supplementary material for: The experiences of a structured pelvic floor rehabilitation program in colorectal cancer survivors with low anterior resection syndrome: A qualitative study
Source: Support Care Cancer. 2026 Jun 26;34(7):697. doi: 10.1007/s00520-026-10892-8 (PMC13309491; doi:10.1007/s00520-026-10892-8)
Supplement: Supplementary file 1 — (DOCX 26.8 KB) [file 520_2026_10892_MOESM1_ESM.docx]

Supplementary File 1 – Low Anterior Resection Syndrome (LARS) score, study-specific satisfaction survey, interview question guide

The survey was a study-specific satisfaction survey developed by the research team with content validated through expert review. Participants rated PFR for psychological impact, self-reported knowledge advancement, and practicality using a 4-point Likert scale. Demographic information and LARS symptom severity were collected during the feasibility project. LARS symptom severity was assessed using the validated 5-item LARS questionnaire. This document contains the Low Anterior Resection Syndrome Score questionnaire, a patient evaluation survey used to assess satisfaction with pelvic floor rehabilitation (overall, psychological impact, knowledge advancement, and practicality), and the interview guide.

**Bowel Function Questionnaire (LARS)**

Name: ____________________________________

Date: _____________________________________

The aim of this questionnaire is to assess your bowel function.

Please tick only **ONE** box for each question.

It may be difficult to select only one answer, as we know the symptoms may vary from day to day. We could kindly ask you to choose one answer which best describes your daily life. If you have recently had an infection or taken any medication that was affecting your bowel function, please do not take this into account and focus on answering questions to reflect your usual daily bowel function.

**Do you ever have occasions when you cannot control your flatus (wind)?**

No, never

Yes, less than once per week

Yes, at least once per week

**Do you ever have any accidental leakage of liquid stool?**

No, never

Yes, less than once per week

Yes, at least once per week

**How often do you open your bowels?**

More than 7 times per day (24 hours)

4-7 times per day (24 hours)

1-3 times per day (24 hours)

Less than once per day (24 hours)

**Do you ever have to open your bowels again within one hour of the last bowel opening?**

No, never

Yes, less than once per week

Yes, at least once per week

**Do you ever have such a strong urge to open your bowels that you have to rush to the toilet?**

No, never

Yes, less than once per week

Yes, at least once per week

**Patient Evaluation Survey**

**Pelvic Floor Rehabilitation Program**

This survey is an opportunity to provide feedback on your experience with the Pelvic Floor Rehabilitation Program associated with Concord Repatriation General Hospital. The Pelvic Floor Rehabilitation Program aims to improve bowel function after bowel cancer surgery and to assist your recovery in returning to normal activities.

You are asked to complete this survey at the conclusion of your rehabilitation program. Your feedback will help us to evaluate and to make future improvements to the service. We appreciate your participation and comments. This feedback may be given anonymously.

Instruction: Please tick the **ONE** box that is most appropriate and fill in the blanks.

1. What is your age in? ___________________________
2. What is your gender? □ Male □ Female □ Intersex
3. What is your main language spoken at home? ___________________________
4. The information given as part of the rehabilitation program was easy to understand.

□ Strongly agree □ Agree □ Disagree □ Strongly disagree

1. The information given during the rehabilitation program was useful.

□ Strongly agree □ Agree □ Disagree □ Strongly disagree

1. The exercise instructions made it easy to continue the exercises at home.

□ Strongly agree □ Agree □ Disagree □ Strongly disagree

1. The information about healthy diet was useful.

□ Strongly agree □ Agree □ Disagree □ Strongly disagree

1. The Pelvic Floor Rehabilitation Program was easy to get to.

□ Strongly agree □ Agree □ Disagree □ Strongly disagree

1. The clinicians were knowledgeable and appropriately answered my questions.

□ Strongly agree □ Agree □ Disagree □ Strongly disagree

1. I was given the opportunity to discuss issues that had never been discussed before with my doctors.

□ Strongly agree □ Agree □ Disagree □ Strongly disagree

1. I received excellent support throughout the program?

□ Strongly agree □ Agree □ Disagree □ Strongly disagree

1. This program has helped me to regain my confidence.

□ Strongly agree □ Agree □ Disagree □ Strongly disagree

1. Overall, how would you rate your experience with the Pelvic Floor Rehabilitation at Concord Hospital?

0 1 2 3 4 5

Very poor Poor Average Good Very good Excellent

□ □ □ □ □ □

1. How could we improve the program?

__________________________________________________________________________________________________________________________________________

**Appendix 15.18: Exit interview**

**Pelvic Floor Rehabilitation Exit Interview Transcript**

Exit interview will be conducted within 1 week after completion of the program via telephone.

Introduction:

Hello I am *[first name and last name, role]* from the University of Sydney. May I please speak to *[participant’s name]*.

If speaking with the correct person: I am calling you to conduct a phone interview because you have recently completed a pelvic floor rehabilitation program of a research project with Carol Chan, physiotherapist. This exit interview is part of the study for the evaluation purpose. Is now a suitable time to talk? [If NO]: Is there another time I can call you back? [If YES]: I am interviewing you to better understand how the pelvic floor rehabilitation program may have impacted your bowel function and day to day life.

Participation in this interview is voluntary and your decision to participate, or not participate, will not affect your relationship with your doctor or ongoing care you receive. This interview will take approximately 15-20 minutes. With your permission, I would like to audio record the interview because I don’t want to miss any of your comments. All information you provide will be kept confidential and only be shared with the research team members. We will ensure the information will not identify you anywhere including in any research reports. You may decline to answer any question or stop the interview at any time and for any reason. Would you have any questions before we start?

May I turn on the recorder?

Just before we begin, do you mind telling me your name.

Interview questions:

First of all, I will start with questions that are related to your bowel symptoms **before** your participation in the pelvic floor rehabilitation program

1. Why did you choose to participate in the Pelvic Floor Rehabilitation Program?

*Prompt: did your surgeon refer you to participate?*

1. What did you expect from the Pelvic Floor Rehabilitation Program before you started the program?
2. How did your bowel symptoms impact your day to day life prior to starting the pelvic floor rehabilitation? *(Illness perception and symptom evaluation)*

*Prompt: How would you describe the severity of your symptoms*

1. How did you manage your symptoms before you participated in the program?

*Prompt:*

1. *Functional self-care strategies include: took exercise (describe type), took medication, increased fibre in diet, took extra fluid, took fibre supplement, changing meal times or skipping meals, avoiding certain food or changing diet or others*
2. *Social activity related self-care strategies e.g. knew the location of a toilet all the time, wore protecting clothing or brought extra underwear when out of the house, wore incontinence pads, planned social events to prevent incontinence*
3. *Alternative self-care strategies e.g. complementary therapies, used spirituality (religion), used trial and error*

*Now I am going to ask you a few questions* ***related to after*** *your participation in the pelvic floor rehabilitation program*

1. Did you notice any change of bowel symptoms after starting the pelvic floor program? If yes, what were the changes? *(after participant answer the first part of the question)* When did they start to change?
2. Now you have completed the Pelvic Floor Rehabilitation, can you suggest if the program has any impact on your day to day life?

*Prompt: Are you are able to do things that you couldn’t do before?*

1. How has it impacted your understanding of bowel symptoms?
2. How has it impacted your self- care management?
3. Was it worth the time for what has changed?
4. In your opinion, what possible changes should be made to the advice or support provided before and after your bowel cancer surgery in regards bowel function?
5. Before we finish, is there anything else you would like to tell us about your experience with this program?

Thank you very much for your time and the information you shared today*.*
