# Supplementary material for: The experiences of a structured pelvic floor rehabilitation program in colorectal cancer survivors with low anterior resection syndrome: A qualitative study
Source: Support Care Cancer. 2026 Jun 26;34(7):697. doi: 10.1007/s00520-026-10892-8 (PMC13309491; doi:10.1007/s00520-026-10892-8)
Supplement: Supplementary file 2 — (DOCX 15.1 MB) [file 520_2026_10892_MOESM2_ESM.docx]

Supplementary File 2
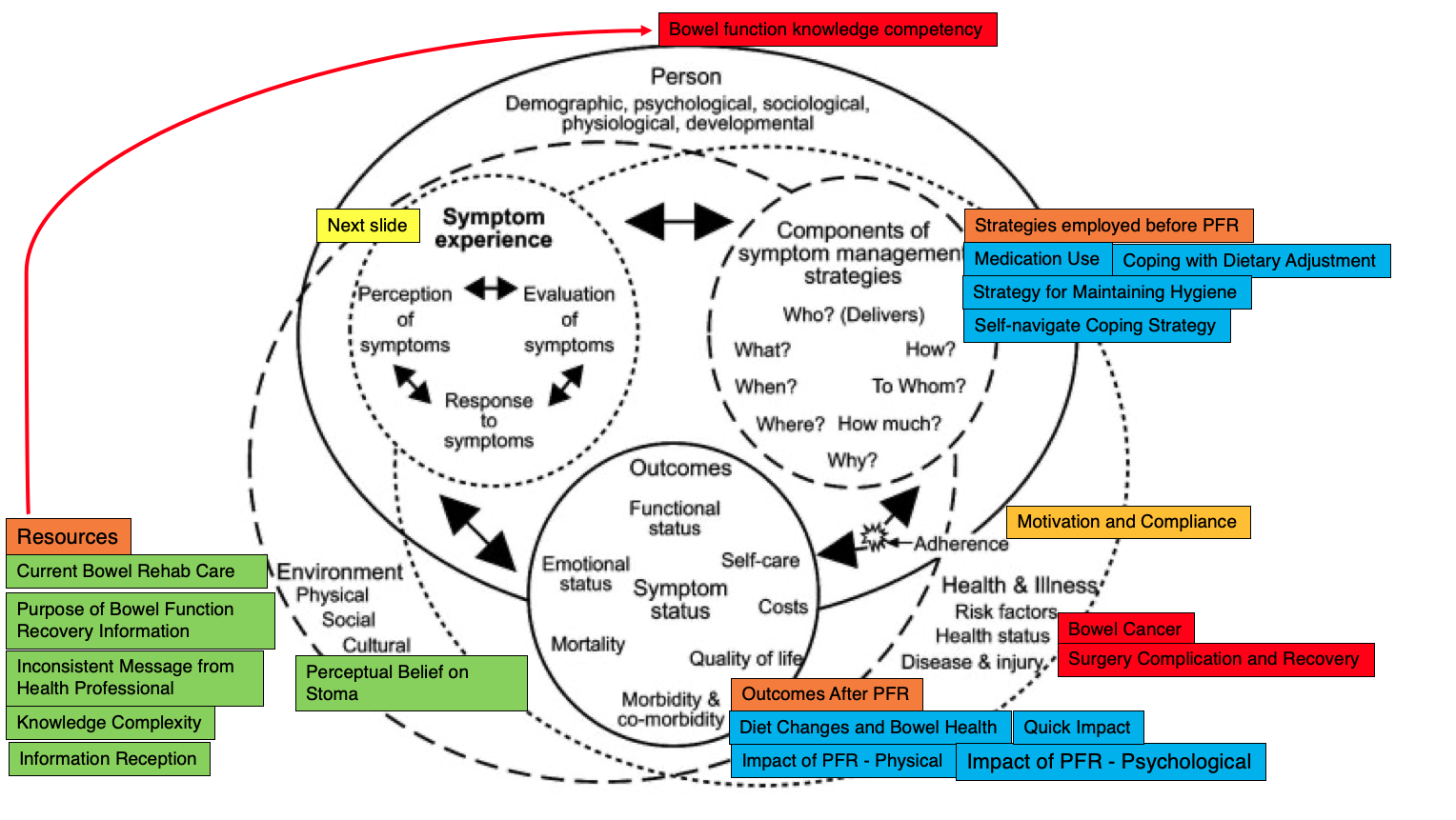


Figure 1. **Symptom Management Theory (SMT)**

Revised Symptom Management Conceptual Model adapted from Dodd, M., Janson, S., Facione, N., Faucett, J., Froelicher, E. S., Humphreys, J., Lee, K., Miaskowski, C., Puntillo, K., Rankin, S., & Taylor, D. (2001). Advancing the science of symptom management. *Journal of Advanced Nursing*, *33*(5), 668–676. https://doi.org/10.1046/j.1365-2648.2001.01697.x

This is an example of visualisation during data analysis using an existing model SMT overlapping the preliminary codes

The codes are coloured to map the COM-B elements-Red: Capability; Orange: Motivation; Green: Opportunity; Blue: Behaviour


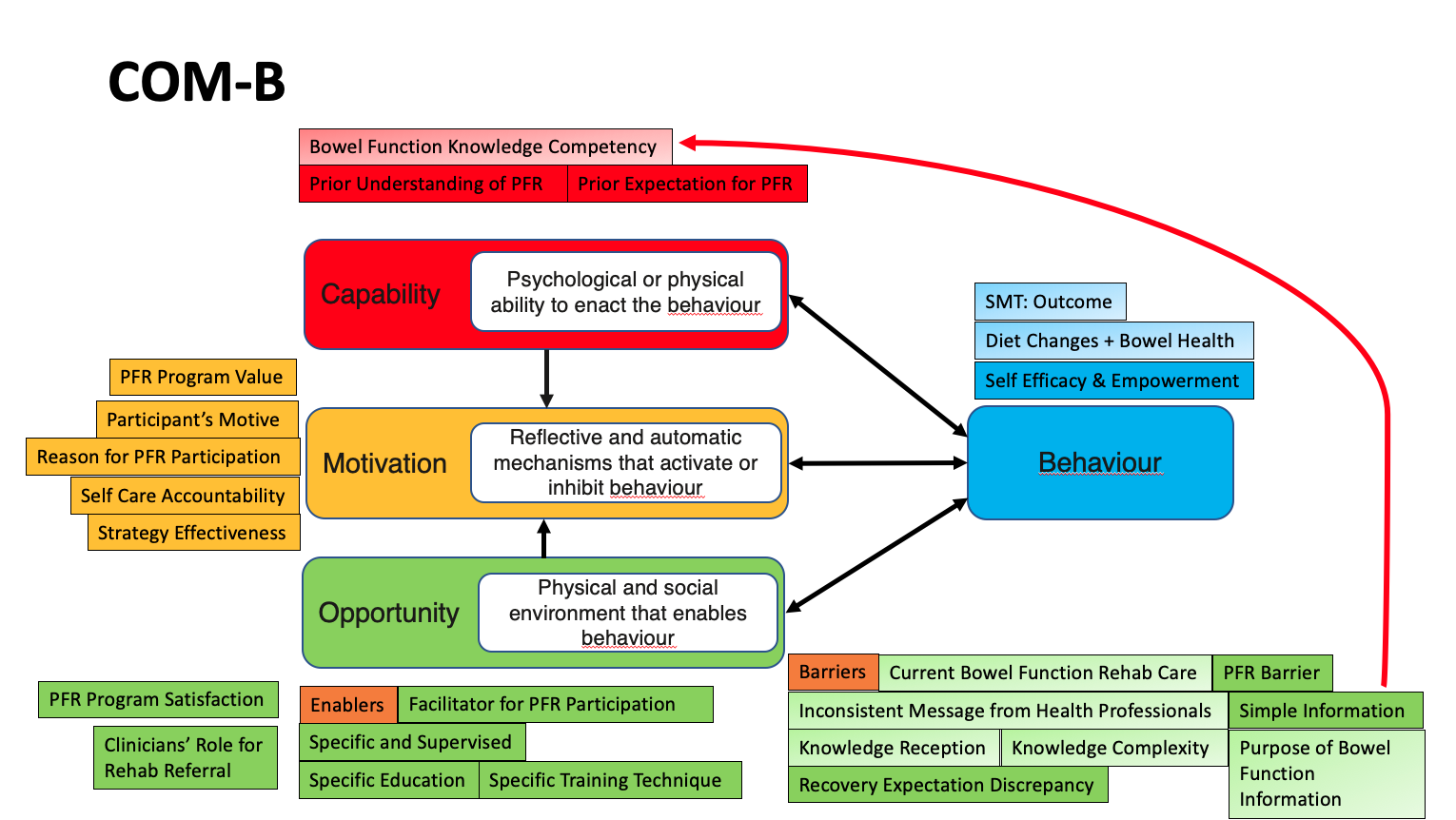


Figure 2. **COM-B Model**

The COM-B system – a framework for understanding behaviour, figure adapted from Michie, S., van Stralen, M.M. & West, R. The behaviour change wheel: A new method for characterising and designing behaviour change interventions. *Implementation Sci* **6**, 42 (2011). https://doi.org/10.1186/1748-5908-6-42

This is an example of visualisation during data analysis using an existing model COM-B overlapping the preliminary codes.

The codes are coloured to map the COM-B elements- Red: Capability; Orange: Motivation; Green: Opportunity; Blue: Behaviour

Codes with the colour gradient have also appeared in the SMT model


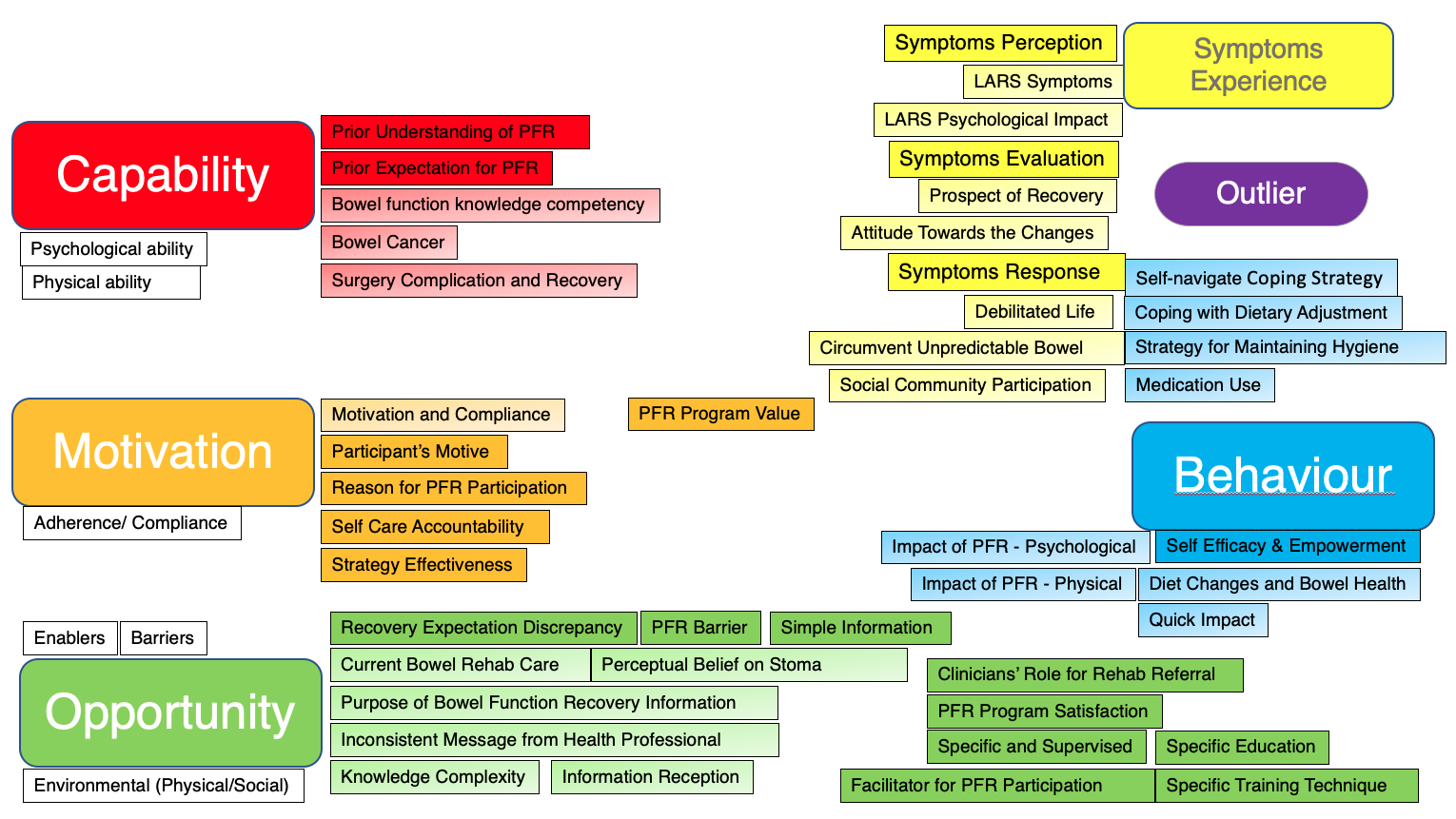


Figure 3. **COM-B + SMT**

The diagram visualised the mapping of preliminary codes, combining with SMT and COM-B.

The codes are coloured to map the COM-B elements- Red: Capability; Orange: Motivation; Green: Opportunity; Blue: Behaviour; Yellow: Symptom experience (SMT)

Codes with the colour gradient have also appeared in the SMT model
