# Supplementary material for: The experiences of a structured pelvic floor rehabilitation program in colorectal cancer survivors with low anterior resection syndrome: A qualitative study
Source: Support Care Cancer. 2026 Jun 26;34(7):697. doi: 10.1007/s00520-026-10892-8 (PMC13309491; doi:10.1007/s00520-026-10892-8)
Supplement: Supplementary file 3 — (DOCX 14.5 KB) [file 520_2026_10892_MOESM3_ESM.docx]

## Supplementary File 3 – Methodological rigour (research team and reflexivity)

Strategies were implemented to minimise potential biases, ensure methodological rigour and support research integrity. An independent researcher experienced in qualitative methods and with no involvement in the PFR intervention conducted the participant interviews. The analysts, KYC and SR, brought topic and methodological expertise respectively, to the interpretation process. Both were actively involved in data analysis, meeting regularly to review coding, interpret findings, and achieve consensus on developing themes. To ensure rigour, both authors (KYC and SR) independently reviewed the codebook and discussed the themes to reach an analytic consensus. The trustworthiness of the analysis and results was established through two types of data sources (surveys and interviews) and adherence to a guiding methodological approach and framework.
