# Supplementary material for: The experiences of a structured pelvic floor rehabilitation program in colorectal cancer survivors with low anterior resection syndrome: A qualitative study
Source: Support Care Cancer. 2026 Jun 26;34(7):697. doi: 10.1007/s00520-026-10892-8 (PMC13309491; doi:10.1007/s00520-026-10892-8)
Supplement: Supplementary file 4 — (DOCX 26.0 KB) [file 520_2026_10892_MOESM4_ESM.docx]

| Supplementary File 4 – Table 1. Additional quotes by subtheme | | | |
| --- | --- | --- | --- |
| Theme | **Subtheme** | | **Illustrative quotations** |
| Living with Unpredictable LARS | **Disruptive, embarrassing, and isolating** **experiences** | | “bowel movements were quite erratic. [with] frequency was up to eight times a day” [P1]  “[I was] missing the toilet many times… [and ended up] soiling my underwear.” [P2]  “embarrassingly enough, I had a couple of accidents… I got to the stage where I was worried about going out, and I would make sure that I was going to a place where there were toilets.” [P5]  “[My friends and daughter tell] me it's in my mind, you know, because first thing I [do when I get somewhere is] go in the toilet.” [P6]  “I would go to the shops, just to get out. And hospital visits and stuff like that. Yeah, but if you are talking about a social life, it was non-existent.” [P9] |
|  | **Understanding of LARS** | **Expectations and realisations of living with LARS** | “I didn't really appreciate how weak the muscles have become [since LARS]… I just thought, ‘oh yes, I'm a perfectly healthy person. I've never had any trouble before. So, you know, why should I be weak [now]?” [P1]  “After some 18 months, I was still having a lot of problems [with bowel function].” [P2]  “Obviously with the condition that I have, [things are] never going to be 100%, [and] assuming at some stage… [I’ll] probably end up having a bag permanently; as you get old, you are more likely [to be] incontinent” [P3]  “They wanted me to have a stoma bag for life, and I said no… I would rather die than have that.” [P9]  “I have no understanding of how the bowels work” [P11] |
|  |  | **Delayed, inconsistent, and absent LARS information** | “I certainly wasn’t [given] much information about the potential for discomfort and the amount of self-care I had to do in order to basically get through every day for quite some time after surgery… it was quite dramatic.” [P1]  “before the surgery I didn't know any of this was going to happen. And I don't think he's worried about that. It's about fixing the problem…. I don’t think you have time to go through [the bowel function] with the busy surgeon to get things… I think that’s what he’s more in tune with and I understand that” [P4]  "I was told at the time by my oncologist it would probably take 3 to 6 months… And that we should get it back. And then hearing the majority of people don't get that, it's, it's a bit to me it's rude and I think they should say, 'all right, this is a percentage of people where it will probably start to work properly, get this larger percentage which you maybe we don't know is an age where your balance won't work properly again." [P6]  "The information is different depending on who you talk to. It's different from the nurse or from the doctor… Everyone needs to get on the same page with everything, you know what I mean?" [P9]  “He just mentioned…it takes some time for the bowel, you know, to get back to normal. But he did say that it will get back to normal.” [P11]  "They give me some information, but not enough… I realise[d] after surgery it’s more complicated than a story [they gave me]." [P12]  “I think if you told people, the extreme of it. People would be very cautious about having surgery.” [P14] |
|  | **Managing in the absence of understanding and guidance** | | “It took quite a few months to get used to… I realised that it’ll never be the same again.” [P5] |
|  |  |  | “I had those pull up underwear on most of the time, [in case] I don’t make it to the loo,” [P5]  “I didn't do anything because I didn't know what to do.” [P11] |
|  |  |  | “Before I used to go out, if I have an appointment or anything to do in the morning. Yeah, I didn't used to have any breakfast at all.” [P6]  “I was taking gastro stop several times a week. Right. Maybe 3 or 4 times a week.” [P14]  “I tried to watch what I eat; you know, I had good days and bad days. I thought I might be alright for one or two days, but I was all over the place.” [P3]  “Even my activities like golf, I wouldn’t be happy about going—would have to prepare myself and take pads.” [P2]  “[Mum] does need to change her daily routine to plan when she goes out, and she needs to make sure she feels empty with her stomach before she can go out... So basically it was just making sure [mum] had emptied her bowel” [P8]  “I would make sure that I was going to a place where there were toilets,” [P5]  “I was experiencing, I would say debilitating [LARS] symptoms and, I wanted to try anything possible that could kind of help with that” [P14] |
| A desire for quality information, timely education, and individualised multimodal support | “And also I think the physio ought to be just part of the process. I don’t know in the future whether [PFR] will be covered by health funds or Medicare. I suppose for some people that might be a concern, the whole post operative care package” [P1]  “I suppose they had a plan, but I think sometimes a program in extended form would help you understand [about LARS].” [P2]  “so preparing somebody for the [stoma] reversal would be a great help. And possibly getting them started on the program like this once the reversal is done, or even before so that people can then become aware of and show what they can do and what potentially is going to happen after the reversal and to prepare them for that.” [P5]  “I wish [there was] funding to continue with this program because it will help patients in a similar situation.’’ [P11] | | |
| Regaining Function and Control through Structured Rehabilitation | **Expectations of pelvic floor rehabilitation** | | “I just don't anticipate…I had nothing to lose” [P1]  “The surgeon [thought] that a referral to [PFR] would basically retrain my bowel with all these exercises… and will be beneficial.”[P3]  “I don’t know what I really expected other than just to learn how to cope better… my surgeon believed it would be a good study for me with the symptoms I am experiencing post-cancer.” [P13] |
|  | **“Back to normal” with structured pelvic floor rehabilitation** | **Understanding my body** | “On the first couple of [sessions], there’s been a dramatic improvement… the pain that was there all the time… it is nowhere near the same kind of horrible intensity that I was going on before this happened. And then eventually [the symptoms] just disappear. So, the first thing you noticed during the lifting the pelvic floor and doing those exercises that take that feeling away a lot quicker.” [P3]  "I understand I'm able to receive pelvic floor exercises, etc. I'm able to complete [them on] my own in my condition. Whereas before I was unable to accept [life] with[out] medication, and now having done this pelvic floor thing, I know that I can control it. I can control it completely. [Symptoms are] not completely solved everything, but I’d say it’s 85-90% better than it was." [P5]  “I feel it has come back to certainly not 100%, but come back to normal,” [P7] |
|  |  |  | **Biofeedback and practical exercises**  “I actually saw the ultrasound and I saw what was happening when I contracted. When I did the pelvic floor exercise, I saw things happening inside of me that spoke major volumes to me.” [P5]  “But then after that, tell me how to breathe. How to do the motion. Then I found that this I... Less go to the toilet and then each time it’s clear.” [P7]  "[I learnt] Not taking care of my diet affects not just my digestion, but also my pelvic floor and muscles. Physio gave me a visual of kind of like the cradle kind of thing and like the pelvic floor and the different layers of everything. So, I understand a lot more about how it works." [P13] |
|  |  |  | **Informed education**  “I don’t know how a layperson would be able to get through it as well as I did know. Because after, after the first or second session talking to [therapist], I gained a greater understanding of how it works. After the first session, I sort of learned about the different muscle groups and the different areas of the muscles, and it was quite enlightening.” [P1]  “One thing is, [the physiotherapist] explained a lot of things to me… and made me more realise what the problem I have and what I should do.” [P12] |
|  |  |  | **Tailored information and management strategies**  "I recognise the things that set me off, like chocolate. Too much, and I have problems. I try to avoid them and only have a little." [P2]  "I used to eat at night, around 8 or 9, but now I just eat around 5:00." [P10]  "We changed something in my diet, and it made a big difference. I don’t really have dairy anymore." [P13]  "I tend to stick to my safe foods when I’m outside the house." [P14] |
|  |  | **More sustainable, confident, and motivated ways forward** | **Functional bowel control**  “I don’t have to think about wearing pads], I've got a stack of pads." [P1]  "I was sure, the muscles get more stronger and more firm because you've been doing those regular exercises, you know, and I'm confident that will go as time goes by." [P3]  “I didn’t have to rush on it,” [P4]  "Basically, [I] have stopped the tablets [immodium] completely." [P5]  "Well, I used to take Movicol every day, but now I hardly use it. Sometimes, I still use it, but I would say maybe once a week." [P12]  “I don’t instantly have to drop everything and run to the bathroom, I’m able to hold my bowels a lot longer… I learned to identify urgency a lot differently.” [P13]  “I feel like I was listening to my body’s cues and almost retraining the muscles, to allow me to hold on for longer.” [P14] |
|  |  |  | **Confidence, comfort, and aspirations**  “I am today without any pull-ups on, I used to use those pants under underpants, but because I have control after the program, because I didn’t have to use them… I feel confident going out now.” [P2]  “It’s a very beneficial program, and [the physiotherapist]’s very passionate about it. So that also helps you because you know that they really believe in it's going to make a difference.” [P4]  “she is not feeling as anxious as before,” [P8]  “Now I can do things more confidently, even plan for travelling to go to travel,” [P11]  “I used to panic… I am able to go out in public and not stress so much. Mentally, I do find now knowing that I've got those exercises and those processes in my toolbox, I'm not panicking.",” [P13] |
|  |  |  | **Motivation**  “You are motivated to keep going” [P2]  “So the next thing I am going to do is join the gym. I’ll be going to start exercising more and more, pelvic exercises at the gym.” [P9]  “That exercise made me feel much better. I told [the therapist], You just led me into that program, and before long, I still needed exercise to keep getting much better—even better now.” [P12] |
