# Supplementary material for: The experiences of a structured pelvic floor rehabilitation program in colorectal cancer survivors with low anterior resection syndrome: A qualitative study
Source: Support Care Cancer. 2026 Jun 26;34(7):697. doi: 10.1007/s00520-026-10892-8 (PMC13309491; doi:10.1007/s00520-026-10892-8)
Supplement: Supplementary file 5 — (DOCX 14.2 KB) [file 520_2026_10892_MOESM5_ESM.docx]

Supplementary File 5 – Symptoms Management Before and After Pelvic Floor Rehabilitation (PFR)

| **Before PFR** | **After PFR** |
| --- | --- |
| Over the counter medication such as anti-diarrheal medication | Maintain the practice of pelvic floor muscle exercises |
| Minimise oral intake: restrict food consumption before going out | Engage in regular functional exercises like running |
| Dietary adjustment: avoid certain food | Diet monitoring: balanced diet and regular meals |
| Pre-emptive bowel emptying | Maintain established bowel routine and toilet habit |
| Planned outings around toilet locations, ensure toilet facility is easily accessible |  |
| Incontinence products such as pull-up underwear, or bring spare clothes |  |
